# Association between Overall Survival and Activities of Daily Living in Patients with Spinal Bone Metastases

**DOI:** 10.3390/healthcare10020350

**Published:** 2022-02-11

**Authors:** Yoshiteru Akezaki, Eiji Nakata, Masato Kikuuchi, Shinsuke Sugihara, Yoshimi Katayama, Haruyoshi Katayama, Masanori Hamada, Toshifumi Ozaki

**Affiliations:** 1 Division of Physical Therapy, Kochi Professional University of Rehabilitation, Tosa 781-1102, Kochi, Japan; akezakiteru@yahoo.co.jp; 2 Department of Orthopaedic Surgery, Okayama University Hospital, Okayama 700-8558, Okayama, Japan; misimaryou0313@gmail.com (H.K.); tozaki@md.okayama-u.ac.jp (T.O.); 3 Department of Rehabilitation Medicine, National Hospital Organization Shikoku Cancer Center, Matsuyama 791-0280, Ehime, Japan; kikuuchi.masato.tu@mail.hosp.go.jp (M.K.); sugihara.shinsuke.rk@mail.hosp.go.jp (S.S.); 4 Department of Rehabilitation Medicine, Okayama University Hospital, Okayama 700-8558, Okayama, Japan; yoshimikatayama@yahoo.co.jp (Y.K.); pjvy4uc1@okayama-u.ac.jp (M.H.)

**Keywords:** spinal bone metastases, radiotherapy, chemotherapy, activities of daily living, overall survival

## Abstract

*Objective:* This study aimed to investigate the association between overall survival (OS) and activities of daily living (ADL) in patients with skeletal-related events. In this study, 265 patients whose clinical parameters were available before radiotherapy were investigated. *Methods:* Age, sex, ADL, pain, the primary site, spinal level of bone metastases, spinal instability, treatment strategy, including chemotherapy or palliative treatment, and OS were investigated. ADL patients with a Barthel index of ≥90 were classified as the high ADL group, while those with a score < 90 were classified as the low ADL group. For OS, patients surviving ≥160 days were classified as the non-poor prognosis group, and those who survived <160 days were classified as the poor prognosis group. *Results:* Age, sex, ADL, pain, the primary site, and treatment strategy for OS were different between the two groups (*p* < 0.1). Logistic regression analysis revealed that ADL, the primary site, and treatment strategy were significant predictors of OS (*p* < 0.05). High ADL, breast cancer, and chemotherapy had a positive effect on OS. *Conclusions:* It is suggested that improvements may be obtained by performing rehabilitation interventions to maintain and improve ADL, by constructing a system for monitoring spinal bone metastases with images before ADL decreases, and by performing interventions such as changes in treatment methods such as RT or surgery at appropriate times.

## 1. Introduction

Approximately one-third of all cancer patients develop bone metastases; breast, prostate, and lung cancer cases account for approximately 80% of all bone metastases [1]. Approximately 70% of patients present metastases involving the vertebral column, commonly at the thoracic and lumbar levels [2]. Bone metastases involve serious complications leading to skeletal-related events (SREs), including bone pain, requiring radiotherapy, pathologic fractures, and spinal cord compression [3,4].

Treatments involve chemotherapy, radiotherapy (RT), surgery, and/or percutaneous procedures (such as vertebroplasty and kyphoplasty) [5]. Radiotherapy is the most common treatment option for bone metastases [6,7,8], effective in reducing symptoms and increasing subjective well-being with minimal side effects [9].

The primary tumor site, good Eastern Cooperative Oncology Group Performance Status (ECOG PS) scale, good Karnofsky Performance Scale, absence of previous chemotherapy and visceral metastasis, single bone metastasis, younger age, and no hypercalcemia were associated with good survival in spinal bone metastases [10,11,12]. However, few reports have examined the role of activities of daily living (ADL) in overall survival (OS) in patients with SREs. Evaluating the factors influencing the prognosis of metastatic spinal disease is essential for providing medical care.

This study aimed to investigate the association between OS and ADL in patients with SREs.

## 2. Methods

### 2.1. Study Design

This retrospective observational study was conducted to investigate OS in patients with spinal bone metastases.

### 2.2. Patients

**Figure 1:**
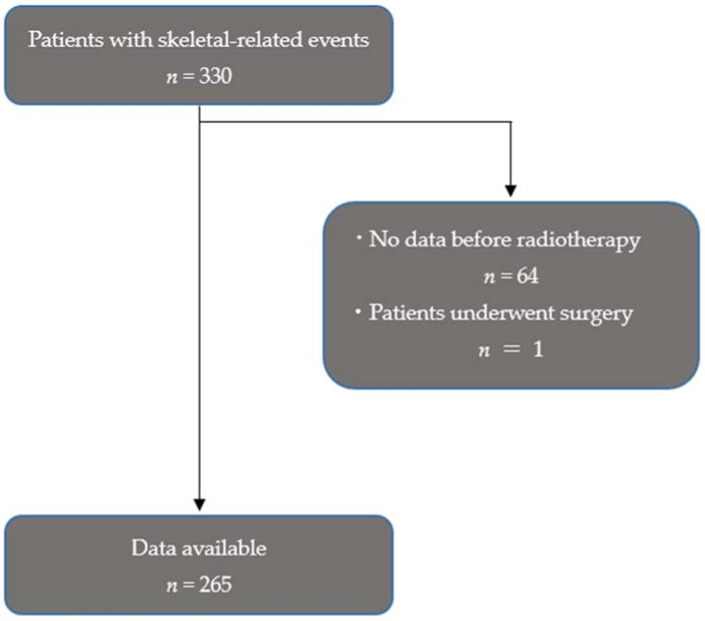
Flowchart of the study.

All patients 18 years and older were included that presented with an SRE and underwent radiotherapy for their SREs at our single tertiary institution in Japan between 2012 and 2016. SRE was defined as bone metastases producing signs and symptoms, such as vertebral bone fracture, painful spinal bone metastases, and spinal cord compression. RT was defined as being performed on spinal bone metastases. Exclusion criteria were: (1) patients that underwent surgery for their SREs; (2) patients in whom there was difficulty assessing the clinical parameters before RT (Figure 1). 

Patients performed muscle strengthening, balance, and ADL exercises from the early stage based on their conditions.

### 2.3. Clinical Parameters

Clinical parameters, including age, sex, comorbidities, ADL, pain, the primary site, brain or visceral metastases, bone metastases other than the spine, spinal level of bone metastases, spinal instability, treatment strategy (chemotherapy or palliative treatment), and OS of all patients were investigated.

### 2.4. Measurement of ADL

The Barthel index is a measure of the ability to perform ADL on a scale of 0–100 (0, very dependent; 100, independent) [13]. The Barthel index assesses the patient’s ability to perform feeding, bathing, grooming, dressing, bowel and bladder control, toileting, chair transfer, ambulation, and stair climbing. The Barthel index was measured by a physical therapist or occupational therapist. The criteria for functional impairment were scores < 90 [14,15]. Patients with a Barthel index of ≥90 were classified as the high ADL group, while those with scores < 90 were classified as the low ADL group. Measurements were estimated before RT. 

### 2.5. Measurement of Pain

Pain in this study was limited to spinal metastasis-related pain and assessed using a numerical rating scale with 0 representing “no pain” and 10 representing “pain as bad as you can imagine.” Pain was evaluated during motion. Measurements were taken before RT. 

### 2.6. The Primary Site and Spinal Level of Bone Metastases 

Breast cancer has a more favorable prognosis than other solid tumors [16]. The primary site was classified into breast and other cancer groups.

Regarding the spinal level of bone metastases, patients with bone metastases in the cervical and thoracic vertebrae were classified as the cervical and thoracic vertebrae group, while patients with bone metastases in lumbar vertebrae were classified as the lumbar vertebrae group.

### 2.7. Measurement of Spinal Instability 

Spinal instability was evaluated using the spinal instability neoplastic score (SINS) [17]. Based on the SINS, patients were classified into three categories: those with stable (SINS, 0–6), potentially unstable (SINS, 7–12), and unstable (SINS, 13–18) spines. Spinal instability was classified into stable and unstable groups. Measurements were recorded before RT. 

### 2.8. Treatment Strategy

Patients who received chemotherapy and palliative treatment after RT were categorized as the chemotherapy and palliative treatment groups, respectively.

### 2.9. OS

Patients who survived ≥160 days were classified as the non-poor prognosis group, and those who survived <160 days were classified as the poor prognosis group. The cutoff value of OS was based on the median value of patients.

### 2.10. Statistical Analyses

Univariate analysis was performed using the chi-squared and Mann–Whitney U tests to identify factors associated with OS. Next, parameters with *p* < 0.1 using univariate analyses were further analyzed using multiple logistic regression analysis to identify the factors that strongly influenced OS.

The relationship between ADL and treatment strategy was analyzed using the Mann–Whitney U test.

Factors affecting OS were assessed by Kaplan–Meier survival analysis, and the log-rank test was used to evaluate differences in survival curves.

All analyses were performed using SPSS software (version 22.0; IBM, Tokyo, Japan). All tests were two-sided, and statistical significance was set at *p* < 0.05.

## 3. Results

### 3.1. Characteristics of the Patients 

The patient characteristics are shown in Table 1. All patients received RT. None of the patients underwent surgery. The study included 143 men and 122 women. The mean age of the patients was 67.2 ± 10.6 (median, 67) years and the Barthel index was 74.7 ± 29.6 (median, 85).

The mean period from the diagnosis of SREs to receiving RT was 3.9 ± 10.0 days (median, 1). SREs of patients progressed after RT. Progression of SREs was confirmed a mean of 149.4 ± 164.1 (median, 119) days after RT.

### 3.2. Univariate Analysis 

The mean OS was 377.5 ± 527.4 (median 158.0) days. The data from the univariate analysis are shown in Table 2. The numbers of patients in the non-poor and poor prognosis groups were 132 and 133, respectively. Age, sex, ADL, pain, the primary site, and treatment strategy for OS were significantly different between the two groups (*p* < 0.1).

The OS values were 836.3 ± 736.7 days and 324.0 ± 506.4 days for the breast and other cancer groups, respectively.

### 3.3. Factors Affecting OS 

The results of multiple logistic regression analysis are presented in Table 3. Multiple logistic regression analysis revealed that ADL, the primary site, and treatment strategy were significant predictors of OS (*p* < 0.05). High ADL, breast cancer, and chemotherapy had a positive effect on OS.

The Barthel indexes were 81.5 ± 25.9 and 67.2 ± 32.1 in the chemotherapy and palliative treatment groups, respectively. The Barthel index was significantly lower in the palliative treatment group than in the chemotherapy group (*p* < 0.05).

**Figure 2:**
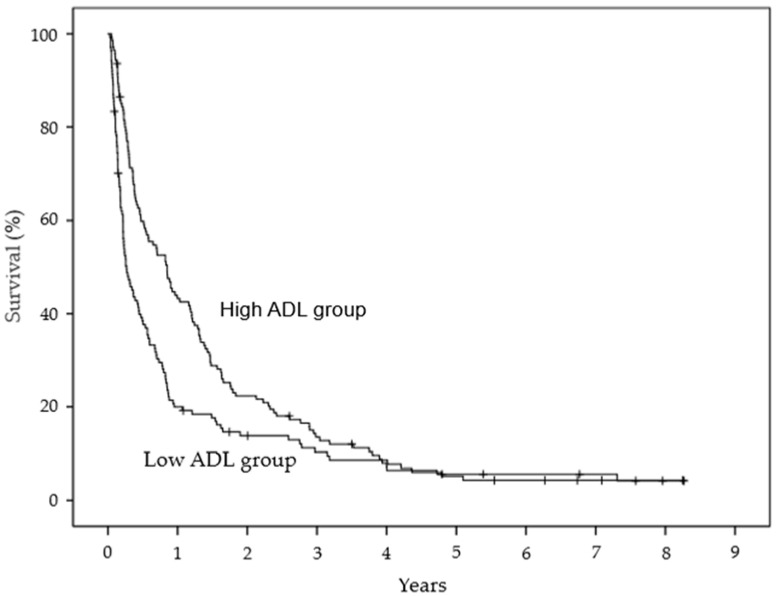
Kaplan–Meier estimates of survival for activities of daily living. ADL, activities of daily living.

**Figure 3:**
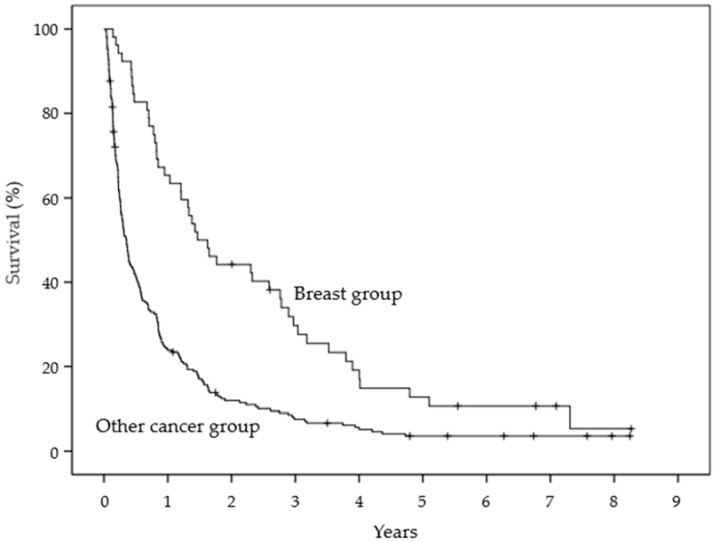
Kaplan–Meier estimates of survival for primary site.

**Figure 4:**
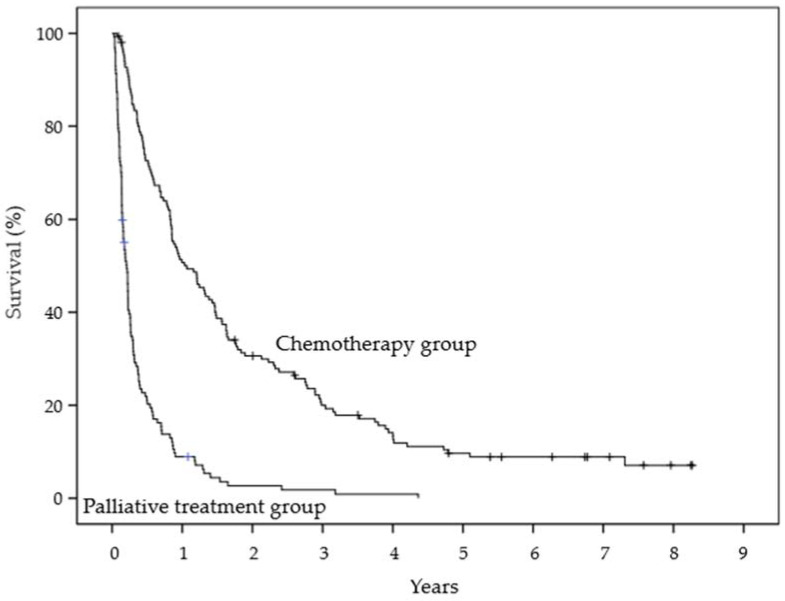
Kaplan–Meier estimates of survival for treatment strategy.

The Kaplan–Meier estimates of survival for ADL at 6 months and 1 year were 59.8% and 43.2% in the high ADL group and 38.4% and 19.9% in the low ADL group, respectively. The groups were significantly different (*p* = 0.001, log-rank test) (Figure 2). The survival estimates at the primary site at 6 months and 1 year were 82.7% and 63.5% in the breast cancer group and 41.5% and 23.9% in the other cancer group, respectively. The groups were significantly different (*p* < 0.0001, log-rank test) (Figure 3). The survival estimates of treatment strategy at 6 months and 1 year were 72.7% and 50.7% in the chemotherapy group and 21.1% and 8.9% in the palliative treatment group, respectively. The groups were significantly different (*p* < 0.0001, log-rank test) (Figure 4).

## 4. Discussion

The purpose of this study was to investigate the association between OS and ADL in patients with SREs. The results showed that ADL, the primary site, and treatment strategy were the best predictors of OS.

The Karnofsky Performance Scale and ECOG PS were reported to be associated with survival in spinal bone metastases [12,18]. Among patients with bone metastases, an ECOG PS score > 2 or a higher serum alkaline phosphatase level had a strong negative effect on the prognosis [10]. In this study, ADL was evaluated using the Barthel index. The Barthel index evaluates not only stroke and orthopedic disorders but also the functional status and ADL of cancer patients [19,20]. In our study, ADL before RT affected the survival rate of patients with spinal bone metastases. Nakata examined the usefulness of a rapid referral system to provide urgent access to MRI scanning, referral to orthopedists, and administration of RT for the urgent management of neurological deficits caused by metastatic spinal cord compression. As a result, the use of the system significantly improved the incidence and severity of neurological deficits following treatment [21]. For the effects of rehabilitation, Rief et al. have shown that guided isometric training of the paravertebral muscles can be safely practiced in palliative patients with stable bone metastases of the vertebral column, improving their pain score and mobility [22]. For patients with SREs, improvements may be obtained by performing rehabilitation interventions to maintain and improve ADL, by constructing a system for monitoring spinal bone metastases with images before ADL decreases, and by performing interventions such as changes in treatment methods such as RT and surgery at appropriate times.

Chemotherapy is useful in prolonging the survival of cancer patients with bone metastasis [23,24,25]. The American Society of Clinical Oncology guidelines recommend that chemotherapy should not be used in patients with solid tumors, exhibiting an ECOG PS score of ≥3 [26]. Some studies have reported that when chemotherapy is administered to patients with poor performance, the response rate of chemotherapy is low, the side effects are strong, and OS is shortened [27,28,29]. In this study, chemotherapy had a positive effect on the survival time of patients with spinal bone metastases. The Barthel index was significantly lower in the palliative treatment group than in the chemotherapy group. Chemotherapy is difficult for patients with low ADL. Therefore, interventions of healthcare professionals are required to prevent ADL lowering in patients receiving chemotherapy. 

OS of patients with bone metastases is shorter than that of patients without bone metastases [10]. The OS rates of spinal bone metastases from breast cancer after 1, 2, and 5 years were 84.8%, 66.3%, and 50%, respectively [16]. The OS rates of bone metastases from lung cancer after 1, 2, and 5 years were 39.8%, 18.7%, and 3.6%, respectively [12]. Breast cancer has a more favorable prognosis than other solid tumors, such as lung cancer [16]. In this study, breast cancer also had a positive effect on survival compared to other cancer types. Thus, the results of our study are in line with those of previous studies.

This study had a few limitations. First, this retrospective study includes various biases such as sample size, patient age, and target disease. Second, physical functions, such as muscle strength, skeletal muscle mass, and balance, were not evaluated; therefore, the effects of these functions on the survival time could not be estimated. Further research is required to examine these factors.

## 5. Conclusions

ADL, the primary site, and treatment strategy were found to be significant predictors of OS in patients with spinal bone metastases who received RT. For patients with SREs, improvements may be obtained by performing rehabilitation interventions to maintain and improve ADL, by constructing a system for monitoring spinal bone metastases with images before ADL decreases, and by performing interventions such as changes in treatment methods such as RT or surgery at appropriate times.

## Figures and Tables

**Table 1:** Characteristics of the patients with bone metastases included in this study.

Characteristic	Number
Primary cancer site	
Lung	94
Breast	48
Prostate	30
Colorectal	23
Stomach	15
Liver	12
Pancreatic	8
Others	35
ECOG PS	
0	3
1	90
2	68
3	67
4	37
Radiotherapy dose	
8 Gy	5
20 Gy	47
24 Gy	1
25 Gy	2
25.2 Gy	1
27 Gy	1
29 Gy	1
30 Gy	179
32.5 Gy	1
36 Gy	1
37.5 Gy	1
39 Gy	1
40 Gy	23
50 Gy	1
Skeletal-related events	
Vertebral bone fracture	194
Painful spinal bone metastases	52
Spinal cord compression	19

ECOG PS, Eastern Cooperative Oncology Group Performance Status.

**Table 2:** Comparison of variables between the non-poor prognosis and poor prognosis groups.

Variable	Non-Poor Prognosis Group	Poor Prognosis Group	*p*-Value
Age (y)	66.0 ± 9.2	68.4 ± 11.8	0.051
Sex (*n*)			0.014
Male	61 (46%)	82 (62%)	
Female	71 (54%)	51 (38%)	
Comorbidities (*n*)			0.220
Yes	59	70	
No	73	63	
ADL (*n*)			0.002
High ADL group	78 (59%)	53 (40%)	
Low ADL group	54 (41%)	80 (60%)	
Pain (scores)	2.7 ± 3.2	3.8 ± 3.7	0.023
Primary site (*n*)			*p* < 0.0001
Breast cancer group	42 (32%)	6 (5%)	
Other cancer group	90 (68%)	127 (95%)	
Brain or visceral metastases (*n*)			0.110
Yes	56	70	
No	76	63	
Bone metastases other than the spine (*n*)			0.388
Yes	56	64	
No	76	69	
Spinal level of bone metastases (*n*)			0.320
Cervical and thoracic vertebrae group	73 (55%)	82 (62%)	
Lumbar vertebrae group	59 (45%)	51 (38%)	
Spinal instability (*n*)			0.175
Stable group	65 (49%)	54 (41%)	
Unstable group	67 (51%)	79 (59%)	
Treatment strategy (*n*)			*p* < 0.0001
Chemotherapy group	104 (79%)	35 (26%)	
Palliative treatment group	28 (21%)	98 (74%)	

ADL, activities of daily living.

**Table 3:** Factors affecting overall survival.

Variable	B	Standard Error	Odds Ratio (95% CI)	*p*-Value
Age	0.017	0.015	1.017 (0.987–1.048)	0.272
Sex	−0.093	0.342	0.911 (0.466–1.780)	0.785
ADL	0.635	0.321	1.887 (1.005–3.543)	0.048
Pain	−0.046	0.045	0.955 (0.874–1.044)	0.313
Primary site	1.743	0.558	5.714 (1.914–17.062)	0.002
Treatment strategy	2.115	0.321	8.286 (4.421–15.531)	*p* < 0.0001

B, unstandardized coefficient; CI, confidence interval; ADL, activities of daily living.

## Data Availability

The data presented in this study are available on request from the corresponding author. The data are not publicly available due to participants’ personal information.
